# Nanocomposite Hydrogel Produced from PEGDA and Laponite for Bone Regeneration

**DOI:** 10.3390/jfb13020053

**Published:** 2022-05-04

**Authors:** Leila S. S. M. Magalhães, Danielle B. Andrade, Roosevelt D. S. Bezerra, Alan I. S. Morais, Francilio C. Oliveira, Márcia S. Rizzo, Edson C. Silva-Filho, Anderson O. Lobo

**Affiliations:** 1 LIMAV—Interdisciplinary Advanced Materials Laboratory, PPGCM—Materials Science and Engineering Graduate Program, UFPI—Federal University of Piaui, Teresina 64049-550, Brazil; leilassmoreira@hotmail.com (L.S.S.M.M.); daniellebenigno@ifpi.edu.br (D.B.A.); alanicaro@gmail.com (A.I.S.M.); marciarizzo@ufpi.edu.br (M.S.R.); edsonfilho@ufpi.edu.br (E.C.S.-F.); 2 Federal Institute of Education, Science and Technology of Piauí, Teresina-Central Campus, IFPI, Teresina 64000-040, Brazil; rooseveltdsb@ifpi.edu.br; 3 Centro Universitário UNINOVAFAPI, Teresina 64073-505, Brazil; franciliooliveira@uninovafapi.edu.br

**Keywords:** nanocomposite hydrogel, laponite, PEGDA, bone regeneration

## Abstract

Herein, a nanocomposite hydrogel was produced using laponite and polyethylene-glycol diacrylate (PEGDA), with or without Irgacure (IG), for application in bone tissue regeneration. The nanocomposites were characterized by X-ray diffraction (XRD), Fourier-Transform infrared spectroscopy (FTIR), and thermal analysis (TG/DTG). The XRD results showed that the crystallographic structure of laponite was preserved in the nanocomposite hydrogels after the incorporation of PEGDA and IG. The FTIR results indicated that PEGDA polymer chains were entangled on laponite in hydrogels. The TG/DTG found that the presence of laponite (Lap) improved the thermal stability of nanocomposite hydrogel. The toxicity tests by *Artemia salina* indicated that the nanocomposite hydrogels were not toxic, because the amount of live nauplii was 80.0%. In addition, in vivo tests demonstrated that the hydrogels had the ability to regenerate bone in a bone defect model of the tibiae of osteopenic rats. For the nanocomposite hydrogel (PEGDA + Lap nanocomposites + UV light), the formation of intramembranous bone in the soft callus was more intense in 66.7% of the animals. Thus, the results presented in this study evidence that nanocomposite hydrogels obtained from laponite and PEGDA have the potential for use in bone regeneration.

## 1. Introduction

Several techniques have been studied to treat bone defects, including autografts and allografts. However, the disadvantages of these techniques include the scarcity of donors, immunogenic rejection, and the possibility of complications related to the disease. Thus, several new biomaterials have been developed through bone tissue engineering to act in bone formation and regeneration. For these biomaterials to be used in bone regeneration, it is necessary to create an environment with properties similar to those of the extracellular matrix of natural bone and bone structures. In addition, these materials must have bioactive factors that provide for the production of new bones and allow their degradation after formation [1].

Thus, several scaffold materials have been developed to be used as three-dimensional supports for bone tissue repair. For example, a study showed that nano-hydroxyapatite (n-HA)/glycol chitosan (G-CS)/hyaluronic acid (HyA) hydrogels were cytocompatible and nontoxic, with excellent potential for application in bone tissue engineering [2]. Another study with thermosensitive methylcellulose (MC) hydrogels containing bassorin (Ba) and halloysite nanotubes (HNTs) achieved excellent proliferation, differentiation, cellular mineralization, and bone cell-specific gene expression [3]. A study produced nanocomposite hydrogels of chitosan, modified halloysite nanotubes, and icariin, and found that the biocompatibility improved the in vivo bone formation and the differentiation of encapsulated cells [4].

The hydrogels (characterized by their three-dimensional networks of various polymers) are optional candidates for scaffolds due to their similarity to the extracellular matrix and the ease of the permeation of oxygen, nutrients, and water-soluble metabolites. Furthermore, bioactive compounds can be easily and safely incorporated into the hydrogel matrix. However, hydrogels have unfavorable characteristics for bone regeneration, such as insufficient mechanical properties and low osteogenic capacity [5,6]. Therefore, the search for hydrogels with high mechanical strength, good mineralization capacity, and antibacterial effects has been the focus of studies. Several materials have been added to hydrogels to improve their properties for bone regeneration. The materials include clays, calcium phosphates, silica, polymers, nanocomposites, etc. [5,6,7]. Clay nanocomposite hydrogels can be used to improve the mechanical performance of conventional hydrogels by incorporating nano-silicates into the nanocomposite hydrogen layers [5,8].

Laponite (Lap, Na_0.7_^+^[(Mg_5.5_Li_0.3_)Si_8_O_20_(OH)_4_]_0.7_^-^) is one of the clays that can be used to produce these hydrogels [8,9]. This nanoclay is a synthetic smectic with 25–30 nm diameter of and 1 nm thickness [10]. Lap crystals have positive charges on the edge and negative charges on the top and bottom surfaces [10,11,12]. In addition, the presence of divalent cations, such as Mg^2+^, in laponite discs promotes better cell adhesion, leading to cell differentiation and, consequently, stimulating osteogenic differentiation [8]. Recent studies have combined Lap with polyethylene-glycol diacrylate (PEGDA) to improve the mechanical and osteogenic capacities of nanocomposite hydrogel, compared to non-clay hydrogels [5,13]. PEGDA is a polyethylene glycol (PEG) derivative widely used to produce functional hydrogels with other monomers or particles by photopolymerization [5].

Furthermore, in the production of nanocomposite hydrogel, UV radiation can be used to improve their properties [14] and has advantages such as low cost, simple equipment, and a fast reaction rate [15]. Irgacure (IG) is a photoinitiator widely used in this process because it is tolerated by several types of cells and has good efficiency in the production of polymer/clay nanocomposites [16,17]. In this context, more studies need to use PEGDA/Lap hydrogels with and without the IG photoinitiator, so as to analyze their effects on bone regeneration.

To date, no published works have associated IG as a photoinitiator to produce Lap/PEGDA nanocomposite hydrogels or evaluated their efficiency to improve bone regeneration. Thus, herein, (i) we produce nanocomposite hydrogels using Lap and PEGDA polymer, with or without incorporated IG; (ii) we characterize the produced nanocomposite hydrogels by X-ray diffraction (XRD), infrared absorption spectroscopy (FTIR), and thermal analysis (TG/DTG); and (iii) we, for the first time, evaluate them in vitro using the *Artemia salina* assay method, and in vivo through a bone defect model of tibias from osteopenic female rats.

## 2. Materials and Methods

### 2.1. Preparation of Laponite Gel

Synthetic silicate nanoplatelets (Laponite^®^ XLG) were purchased from Southern Clay Products, Inc. (Louisville, KY, USA). Lap is a trademark of the company BYK Additives Ltd. Lap (6%) was dispersed in water at room temperature (25 °C). The solution was placed in a mechanical stirrer for 20 min at a speed of 3000 rpm until a clear solution was formed [13,18]. PEGDA (Average Mw: 6000, number 701963-1g, lot #MKCC8234, Sigma-Aldrich) was also diluted to 10% (*m*/*v*). IG 2959 (Sigma-Aldrich, Burlington, MA, USA) was used as a photoinitiator with a concentration of 0.05% of the total mass.

### 2.2. Hydrogel Synthesis

**Figure 1:**
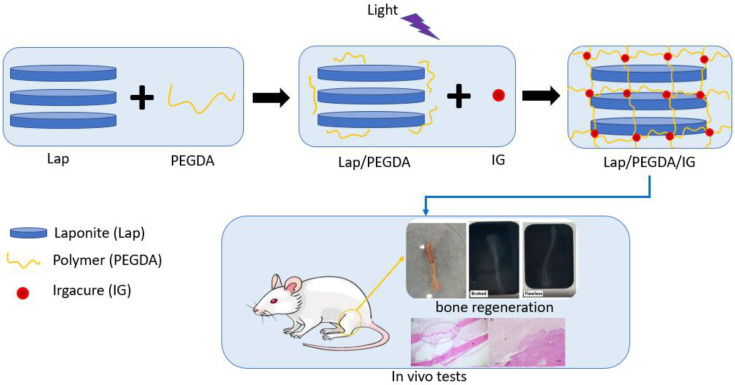
Production scheme of nanocomposite hydrogels.

Four groups were used for the in vitro assay, as shown in Table 1. In group one, 6% Lap was used. In group two, the composite with 6% Lap and 10% PEGDA was used, which was light-cured for 5 min with UV irradiation (365 nm) at 45 µWm^−2^ (Blak-Ray Model XX-15L UV Lamp); the PEGDA solution was added to a falcon tube already containing the laponite gel. The tube was placed under mechanical agitation (3000 rpm, for 20 min), and the hydrogel was placed under a UV light. Group three contained 6% Lap, 10% PEGDA, and IG 2959 0.05% photoinitiator, which was light-cured using UV irradiation (365 nm) at 45 µWm^−2^ (Blak-Ray Model XX-15L UV Lamp) for 5 min; the production methodology of this hydrogel was similar to that of group two, the only change being the addition of the IG solution in this procedure. In group four, only 10% PEGDA was used, which was also photopolymerized using UV irradiation (365 nm) at 45 µWm^−2^ for 5 min [13,18]. Figure 1 presents a production scheme for the nanocomposite hydrogels.

### 2.3. Characterization of Hydrogels

Before being characterized, the hydrogels were lyophilized by freezing them at a temperature of −80 °C and then subjecting them to vacuum, which removed the water by sublimation. Freeze drying was performed on the LS3000 Freeze Dryer. Scanning electron microscopy (SEM) images were captured using a Quanta FEG 250-FEI microscope (10–40 kV). Before analyses, a thin layer of gold (~10 nm) was deposited using a sputtering machine (Quorum Company, argon plasma at 2 × 10^−1^ mbar, and a current of 30 mA, for 2 min). FTIR (Fourier transform infrared spectroscopy) spectra were acquired on a PerkinElmer Spectrum 100, KBr window, 4 cm^−1^ resolution, and 16 scans. The samples were diluted in KBr, in a proportion of 1:20 mg (material:KBr), macerated in agate grain, formed into pellets, and analyzed. XRD was performed in a Shimadzu XDR-6000 diffractometer with an angular variation of 5–100° (2θ), CuKα radiation (λ = 1.5418 Å), voltage of 40 KV/30 mA. Thermogravimetric analysis was performed using TA Instrument SDT Q600-0883 (DSC-TGA) equipment, with heating rate of 10 °C min^−1^, flow rate of 100 mL·min^−1^, under argon atmosphere, using a crucible of alumina. Analyses were performed between 20 and 1000 °C, with approximately 5 mg of each sample.

### 2.4. In Vitro Tests

The acute toxicity of the developed systems was evaluated from tests with *Artemia salina* [19]. For that, *Artemia salina* (Maramar Pet) eggs were incubated in artificial seawater prepared from sea salt using 1.0 L of distilled water, 15.153 g of NaCl (Dinâmica), 1.398 g of MgCl_2_ (Impex), 1.888 g of MgSO_4_ (Isofar), 0.652 g of CaCl_2_ (Dinâmica), 0.414 g of KCl (Dinâmica), and 0.116 g of NaHCO_3_ (Sigma-Aldrich) [20]. This test was prepared at concentrations of 0.1; 0.5; 1.0, and 5.0 mg·mL^−1^ in 10 mL of artificial saline solution, and then 10 nauplii of *Artemia salina* was added to each container. Soon after, the hydrogels were placed in contact with these solutions. All tests were performed in triplicate and toxicity was determined according to the amount of *Artemia salina* alive after 24 h and 48 h of contact. During the tests, the lighting of the systems was maintained under controlled conditions, and the negative control was conducted using synthetic saline solution.

### 2.5. In Vivo Tests

The in vivo tests were carried out in accordance with the registration for research ethics committee n° 002P-V2-2019. Fifty female Albinus rats of Wistar lineage and initial weights of 250 to 300 g were used. These were housed in the Uninovafapi vivarium. They were kept in an acclimatized room, in a photoperiod of 12 h light/12 h dark, in collective cages (four animals/box) with standard rat food (Labina) and free access to water. Twenty-five female rats were randomly assigned, as shown in Table 2.

Analyzes of bone mineralization evolution were performed by histological analysis 45 days after implantation [21,22].

#### 2.5.1. Protocol and Surgical Procedures for Oophorectomy

Oophorectomy was performed with the female rats under intramuscular anesthetic of ketamine and xylazine [23,24]. A 1.5 cm-long median incision was made in the subcutaneous skin on the animal’s back, below the last rib. The ovaries were exposed and removed to obtain material. The animals were at rest for a period of 45 days, which was sufficient time for the appearance of osteopenia [24,25]. Surgical preparation was performed on the right tibia of the rats, with trichotomy of the region to be incised and asepsis with iodine. Surgical procedures were common for all animals, with the implantation of all systems containing laponite and PEGDA, according to the groups mentioned above, on the right tibia of each animal. Preparation for implantation was completed with the aid of a trephine-type drill and a surgical micromotor with abundant saline solution irrigation, to produce an elliptical bone defect until r the medullary canal, where it was filled in with the materials mentioned above. After 45 days of biomineralization, the animals were anesthetized and sacrificed with excessive doses of sodium thiopental intraperitoneally [24,25].

#### 2.5.2. Histological Analysis

The right tibias were removed and radiographed in a dental X-ray machine with Kodak periapical films, to see if the perforation was adequate, and then fixed with 10% formalin solution. After fixation, the tibias were placed in an aqueous solution of sodium hydroxide and EDTA (ethylenediamine tetraacetic); then, these were hemisectioned towards the cut surface, in a paraffin block, and subjected to routine histological technique. Eight cuts were made from each block, approximately 5 mm-thick and 15 mm between each level, stained with hematoxylin and eosin, and analyzed under a microscope. Histological sections were obtained by dissecting the tibiae, fixed in a 10% formalin solution and decalcified in a 10% ethylenediamine tetraacetic acid (EDTA) solution, pH 7.0, for 30 days, and then dehydrated with alcoholic solutions (70 to 100%), diaphonized in xylene, and embedded in paraffin blocks. Soon after, sections were made in a microtome with a thickness of 5 µm, and were stained with hematoxylin and eosin stains (H.E.). Sections were analyzed on an Opticam O500R light microscope at 4×, 10×, 20×, and 60× magnifications by a pathologist who was blinded to the study groups. Images were recorded using a 5.3-megapixel resolution camera (UltraK HD Opt5003 Optifocus). The qualitative analysis of the sections was performed by measuring the cortical photomyography of the injured bones using the software Opticam OPTHD 3.7 (Opticam Inc., Technology/Microsoft Windows, Andover, MA, USA). Histological slides were assessed by the semi-quantitative system of analysis, considering 0–3 scores for the fibrocartilage process, endochondral ossification, bone spicules, and intramembranous ossification. The intensity of changes was semiquantitatively evaluated and denoted as follows: (−) no changes; (+) mild change; (++) moderate change; (+++) intense change. The results obtained are expressed in terms of cumulative scores (0 to 12) [26].

### 2.6. Statistical Analysis

The statistical data were analyzed by analysis of variance (ANOVA) and the Tukey test, with Student–Neuman–Keuls as a post hoc test, using the program GraphPad Prism© version 6.00 for Windows (GraphPad Software, San Diego, CA, USA). Nonparametric statistical analysis (double-way ANOVA) was used for histological evaluation. Data were expressed as mean ± S.E.M. The control groups were compared with the other groups, with a statistically significant difference for *p* ≤ 0.05. [24,25].

## 3. Results and Discussion

### 3.1. Characterizations

The materials analyzed by the different techniques are samples of freeze-dried structures, free from moisture. The hydrogel can be reconstituted by adding water, to ensure a good characterization of the material structure without the influence of water.

**Figure 2:**
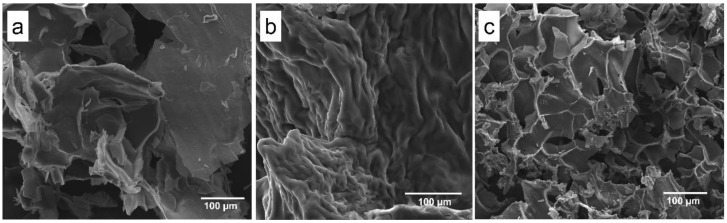
Scanning electron microscopy images collected from the (**a**) Lap 6%, (**b**) PEGDA 10% + Lap 6%, and (**c**) PEGDA 10% Lap 6% + IG 0.05% groups. All images were collected using 500× magnification.

SEM images (Figure 2) displayed a lamellar structure (Lap 6%, Figure 2a) that is typically observed with Lap, a roughness structure of PEGDA 10% + Lap 6% (Figure 2b), and a porous PEGDA 10% + Lap 6% + IG 0.05% nanocomposite hydrogel morphology (Figure 2c) after crosslink. The rough morphology is typically observed for PEGDA hydrogels; however, some lamellar honeycomb-like structures were shown when Lap was added (Figure 2c). A porous honeycomb structure is considered very interesting to apply as scaffolds for bone tissue engineering, as previously reported by [7,27,28,29,30,31,32].

**Figure 3:**
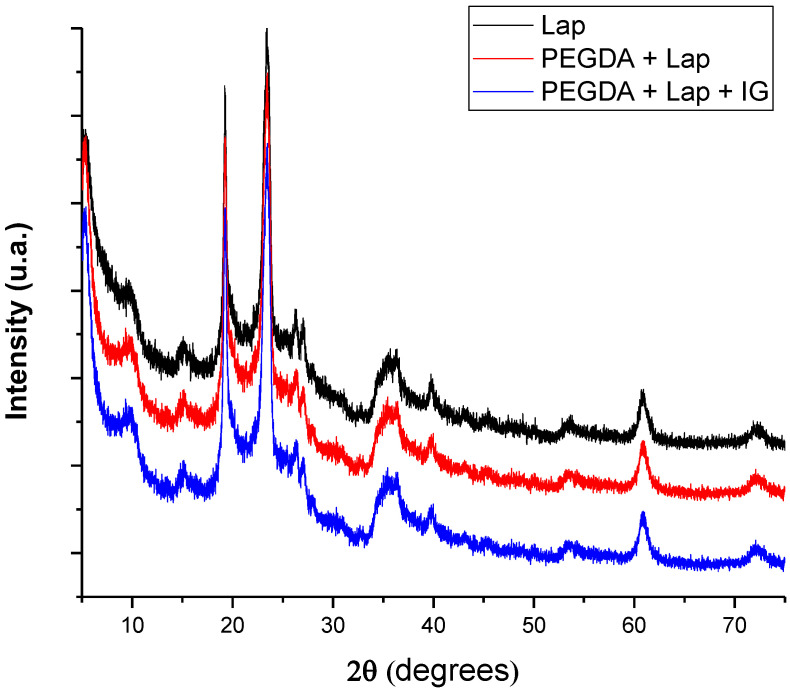
X-ray diffractograms of lyophilized nanocomposite hydrogels.

Characterization using XRD allows for the microstructural evaluation of the proposed systems, whose diffractograms are in Figure 3. The pattern of diffraction reflections of Lap is located at 2θ = 5.38°, 9.53°, 19.20°, 23.52°, 26.30°, 27.16°, 35.77°, 39.95°, 53.58°, 60.90°, and 72.51° [27,28]. Reflections of Lap referring to 2θ = 5.38°, 19.20°, 27.16°, 35.77°, and 60.90° are related to crystallographic planes (001), (100), (005), (110), and (300), respectively [29,30,31]. After the incorporation of PEGDA and IG in the Lap structure, the nanocomposite hydrogel presented the same crystallographic patterns as pure laponite. In addition, reflections or the displacement of reflections referring to pure laponite did not appear. These results indicate that the crystalline structure of Lap is preserved in the nanocomposite hydrogel, and that PEGDA and IG are incorporated into the clay surface [32,33,34,35].

**Figure 4:**
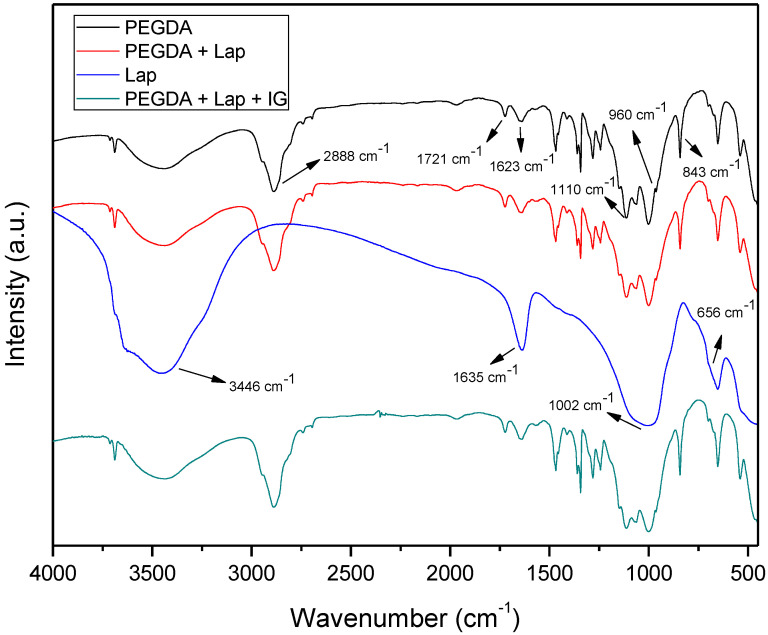
FTIR spectra for Lap, PEGDA, and nanocomposite hydrogel.

The FTIR spectra of Lap, PEGDA, and nanocomposite hydrogels are presented in Figure 4. The PEGDA spectrum includes the bands at 2889, 1721, and 1623 cm^−1^, which refer to the asymmetric stretch vibration of CH_2_, symmetrical stretching vibrations in acrylates of C=O, and the vibration of the aliphatic double bond C=C, respectively. The bands located at 1110, 960, and 843 cm^−1^ are related to C–O stretch vibration, out-of-plane vibration of the symmetrical stretching of CH_2_=CH, and symmetric stretch vibration of CH_2_=CH [36,37,38,39]. The Laponite spectrum has bands at 3436, 1635, 1002, and 656 cm^−1^, which are related to intramolecular stretch vibrations of OH from adsorbed H_2_O, OH bending vibrations, Si–O stretch vibrations, and Mg–OH–Mg bending vibration, respectively [36]. The spectra of the nanocomposite hydrogel (PEGDA + Lap and PEGDA + Lap + IG) show the characteristic bands of PEGDA; however, the clay bands appear reduced and masked by the polymer absorption bands. The absorption bands related to the IG photoinitiator also do not appear in the FTIR spectrum of the PEGDA + Lap + IG nanocomposite hydrogel, because these are also masked by the polymer bands. These results indicate that polymeric PEGDA chains are entangled over laponite in the nanocomposite hydrogel [28,40,41,42].

**Figure 5:**
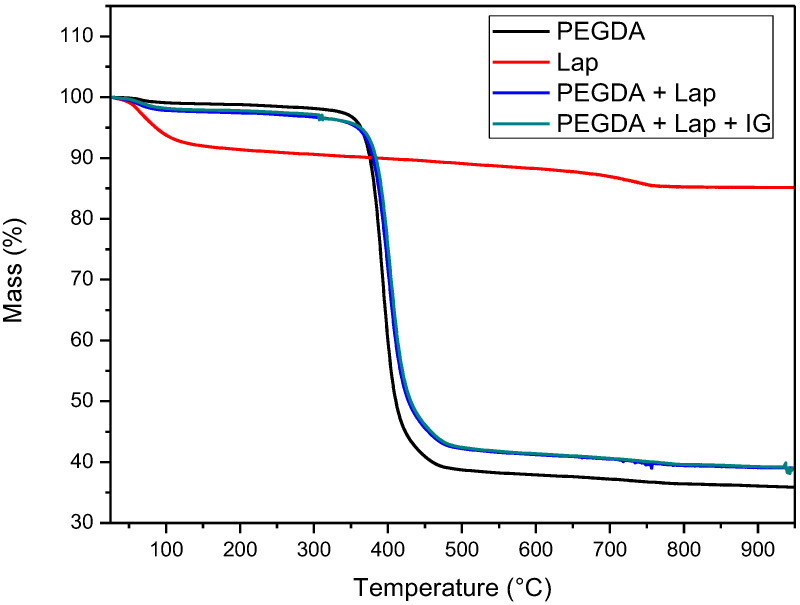
TG Curves of Lap, PEGDA, and nanocomposite hydrogel.

**Figure 6:**
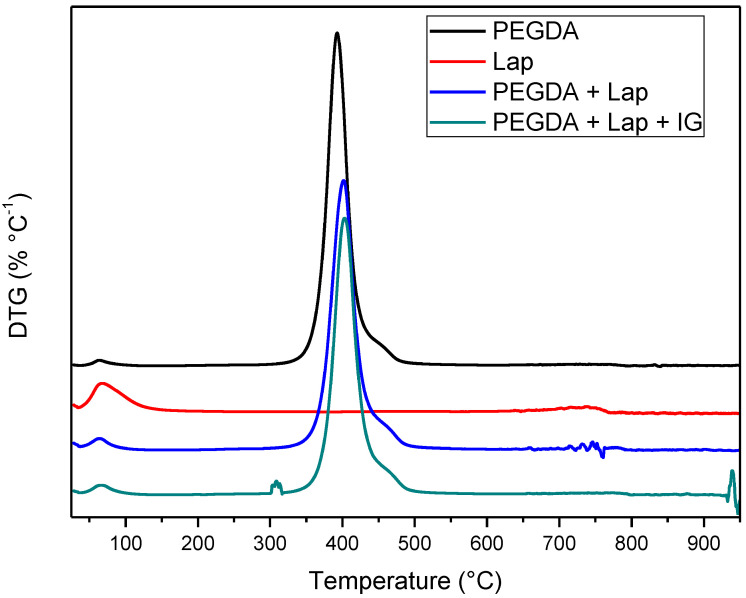
DTG Curves of Lap, PEGDA, and nanocomposite hydrogel.

The thermal stability of laponite, PEGDA, and nanocomposite hydrogel (PEGDA + lap and PEGDA + Lap + IG) was evaluated using TG/DTG curves. The TG/DTG results are shown in Figure 5 and Figure 6. The TG/DTG of PEGDA evidence two stages of mass loss. The first stage occurs with a mass loss of 0.9%, with a maximum temperature of 62 °C, and is related to the loss of residual water. The second stage of degradation occurs with a mass loss of 55.72% and a maximum degradation temperature of 391 °C. This degradation event is related to PEGDA polymer chains [43,44,45,46,47]. On the other hand, Lap has a mass loss stage (8.1% mass loss) at a maximum temperature of 68 °C, related to the evaporation of water, which occurs up to 180 °C. The TG curve also elucidates that laponite has thermal stability up to approximately 690 °C; above this temperature, another stage of thermal degradation (mass loss of 2.3%) refers to the dihydroxylation process of clay layers [48,49].

The TG curves of the nanocomposite hydrogels (PEGDA + Lap and PEGDA + Lap + IG) are similar, showing two stages of degradation. The first stage of degradation of the two nanocomposite hydrogels is related to water loss. The second stage of thermal degradation is related to the degradation of the nanocomposite hydrogel matrix (PEGDA + Lap and PEGDA + Lap + IG). Furthermore, the second stage of thermal degradation of the nanocomposite hydrogel showed that the maximum degradation temperature was displaced due to the presence of Lap (PEGDA + Lap) (400 °C) and Lap/IG 2959 (PEGDA + Lap + IG) (402 °C) in relation to pure PEGDA (391 °C). This result indicates that the presence of laponite in nanocomposite hydrogels improved their thermal stability. This result is confirmed by the amount of final residue in the TG curves, where the nanocomposite hydrogel had higher amounts of residues (PEGDA + Lap = 38.9% and PEGDA + Lap + IG = 39.3%) than pure PEGDA (35.8%) [48,49].

### 3.2. In Vitro Test

**Figure 7:**
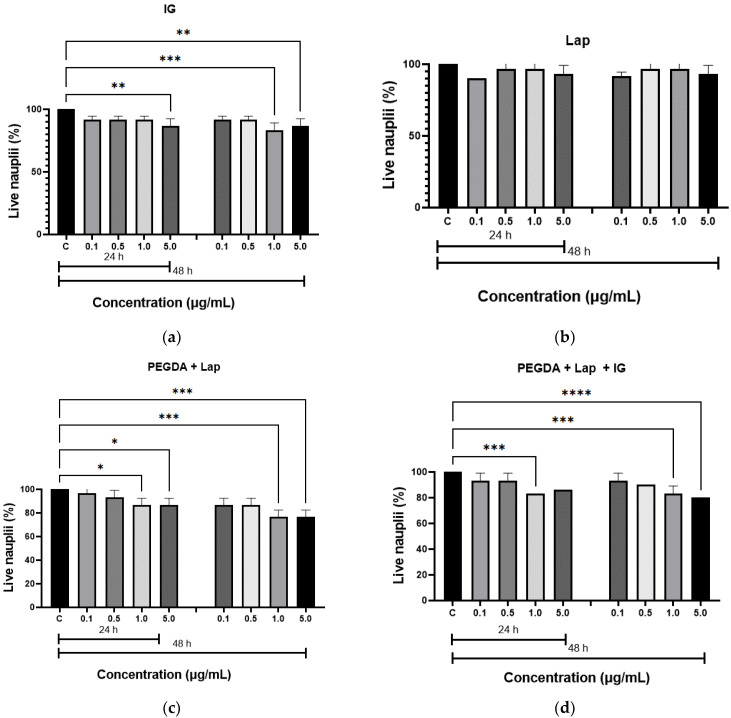
Acute toxicity, by *Artemia salina*, for: (**a**) IG, (**b**) Lap, (**c**) PEGDA + Lap, and (**d**) PEGDA + Lap + IG. The control groups were compared to the other groups, with a statistically significant difference for *p* ≤ 0.05; * = *p* ≤ 0.0303, ** = *p* ≤ 0.0042, *** = *p* ≤ 0.0004, and **** = *p* ≤ 0.0001.

The in vitro toxicity of the hydrogels was evaluated by the *Artemia salina* lethality assay. The results of the toxicity test are illustrated in Figure 7. The hydrogels were not toxic to live nauplii. When comparing the results of the nanocomposite hydrogels with the control group (nauplii cultured in saline solution), the amount of live nauplii was greater than 80.0% after 24 h at all concentrations studied, and greater than 50.0%, after 48 h, which confirms that the samples do not have toxicity. These results confirm the non-toxicity of hydrogels, as described in the literature [20,50].

### 3.3. In Vivo Tests

The histopathological analysis of rodent tibial bone tissue was based on qualitative (descriptive) and semi-quantitative assessments. In all groups studied, no bone deformation was found that could harm the selected methodology. The analysis of histological sections of the samples without defects from different groups (*n* = 3) shows a bone tissue composed of compact lamellar bone (external surface) and cancellous bone, presenting an anastomosing network of mineralized osteoid spicules (trabeculae) randomly arranged near the tip of the bone. The intact periosteum was well-visualized in all samples, in addition to the medullary cavity with blood cells.

**Figure 8:**
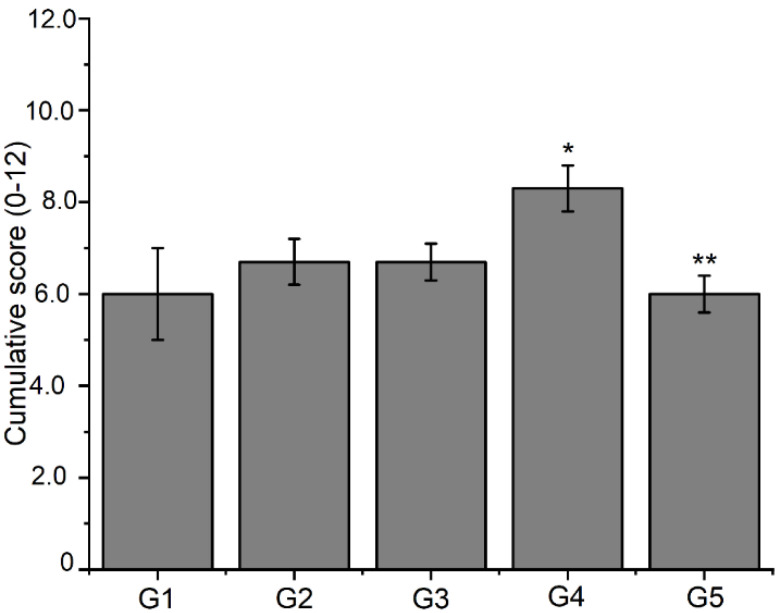
Analysis of histological slides assessed by a semi-quantitative system considering 0–3 scores for each process of fibrocartilage, endochondral ossification, bone spicules, and intramembranous ossification. Data are expressed as mean ± S.E.M of cumulative scores from 0 to 12 (*n* = 3 animals per group). Significance was determined by double away ANOVA followed by Tukey’s test. * *p* < 0.05 compared to the control group (G1) for the intramembranous ossification process; ** *p* < 0.05 compared to the control group (G1) for the fibrocartilage process.

In the histological sections from all experimental groups, bone neoformations were observed in the defect area, with immature, non-lamellar bone trabeculae irregularly distributed. In the new trabeculae, voluminous osteoblasts appear on the periphery of the bone features, and considerable amounts of osteocytes appear within the matrix. The results revealed the adequate and high progression of new bone formation (Table 3 and Figure 8).

**Figure 9:**
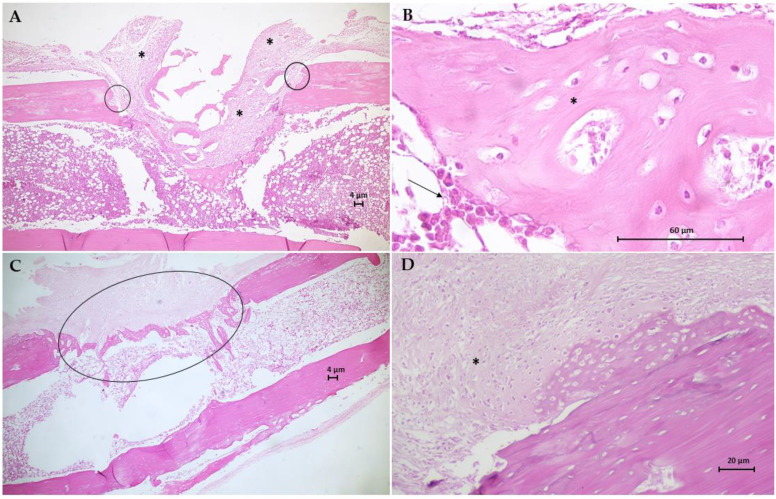
Photomicrographs of bone tissue from G1 group (**A**,**B**) and G2 group (**C**,**D**). Hematoxylin-Eosin (H.E.) stain (Scale bar: 4 µm, 20 µm and 60 µm). (**A**) This longitudinal section shows the formation of a granulation tissue (*) filling the area of the critical defect, anchoring at the extremities of the fractured bone (circles). (**B**) Detail of A showing large numbers of osteoblasts (arrow) surrounds immature and poorly mineralized bone trabeculae (*). (**C**) This section shows the extensive irregular formation of trabecular bone formed by an anastomosing network of bone spicules (circle). (**D**) Longitudinal section of fibrocartilaginous tissue formation in soft callus (*).

The histological results for the sections of the tibial surgical defect in the control group (G1) are in Figure 9. Animal bone tissue samples demonstrated the formation of a granulation tissue, with proliferation of mesenchymal cells, filling the area of the critical defect, anchoring at the extremities of the fractured bone, and surrounding old bone fragments in reabsorption (Figure 9A). Furthermore, trabeculae tissue from the fusion of bone spicules (immature) developed inside the medullary cavity. Large numbers of osteoblasts are seen surrounding immature and poorly mineralized bone trabeculae (Figure 9B). One of the animals exhibited soft callus fibrocartilaginous tissue, anchored mainly in the compact bone periosteum, with the appearance of endochondral ossification at the site of the critical defect. Plaques of newly formed bone trabeculae from the soft callus have a large number of osteocytes, the same as seen in the newly formed cancellous bone at the site of the critical defect and with moderate mineralization. Figure 9C,D shows the histological results for the G2 group. Bone tissue samples from this group demonstrate the fibrocartilaginous tissue formation in the soft callus and extensive irregular formation of trabeculae bone formed by anastomosed network of bone spicules oriented perpendicularly to the cortical axis and within the medullary cavity, which fills the entire area of the critical defect. There are still areas of cartilage–bone transformation (endochondral ossification) and poorly mineralized osteoids with many osteocytes.

**Figure 10:**
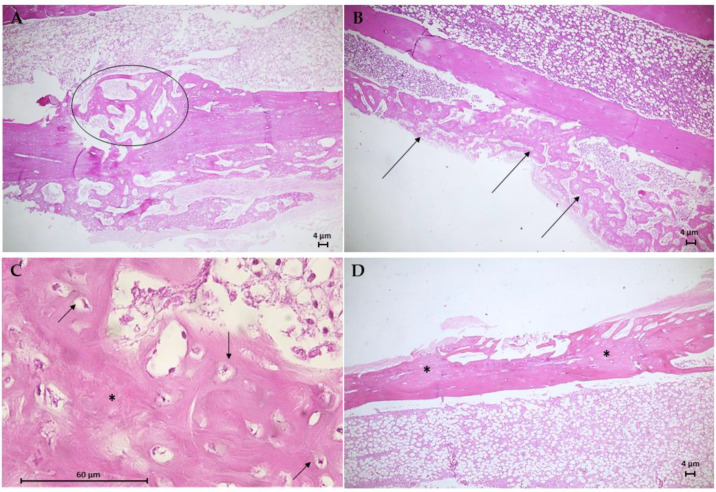
Photomicrographs of bone tissue from the G3 group (**A**), the G4 group (**B**,**C**), and the G5 group (**D**). Hematoxylin–Eosin (H.E.) stain (Scale bar: 4 µm and 60 µm). (**A**) This longitudinal section shows the formation of reconstructive bone tissue in the critical defect (circle). (**B**) Presence of intramembranous ossification in the soft callus (arrows). (**C**) Detail of B showing developing osteons with moderated trabecular mineralization (*) and osteocytes (arrows). (**D**) Longitudinal section of moderate trabecular mineralization filling both ends of the critical defect.

The histological results for the G3 group are in Figure 10. Animal bone tissue samples showed the formation of reconstructive bone tissue in the area of the critical defect by advanced endochondral ossification (Figure 10A), displaying the presence of newly formed spongy bone anchored in preexisting compact bone. Note an osteoid with more mineralized areas invading the fibrocartilaginous callus. An analysis of histological sections of bone tissue samples from the G4 group (Figure 10B,C) revealed the presence of intramembranous ossification within the soft callus, with irregular anchoring observed on the external surface of the preexisting compact bone. The formation of intramembranous bone in the soft callus was more intense in 66.7% of the animals. Few osteoclasts were observed adjacent to the osteoid trabeculae, and no area of bone defect was visible. Figure 10D shows the results of the histological analysis of the G5 group. In bone tissue samples, trabeculae bone filled both ends of the critical defect and with randomized areas of mineralization. In addition, intramembranous ossification on the outer surface of the soft callus stood out.

The regions where laponite composites were placed had higher a density than in the control group, suggesting the capacity for the deposition of neoformation of bone tissue. This is because laponite bioceramic can induce bone formation in vivo [51]. The PEGDA–Lap system is a promising material for applications in bone regeneration therapy, as evidenced in this study, whose results point to an adequate distribution of osteoblasts, as well as the ability to promote the formation of new bone [52].

## 4. Conclusions

Herein, a nanocomposite hydrogel was produced from Lap clay and PEGDA polymer, with or without the IG photoinitiator. SEM images exhibit a lamellar, porous, and honeycomb-like structure from nanocomposite hydrogel. XRD results showed that the crystallographic structure of Lap was preserved in the nanocomposite hydrogel after the incorporation of PEGDA and IG. FTIR results indicated that the PEGDA polymer chains were entangled on the Lap. The characterization by TG/DTG found that the presence of Lap improved the thermal stability of the hydrogels in relation to the pure PEGDA polymer. Artemia salina toxicity tests demonstrated that the hydrogels have no toxicity, because the amount of live nauplii was 80.0% after 24 h and 48 h. The bone healing capacity promoted by nanocomposite hydrogels was demonstrated in vivo in a bone defect model using tibiae from female osteopenic rats. The in vivo results of this study provide evidence of the potential of the developed nanocomposite hydrogel for orthopedic applications.

## Figures and Tables

**Table 1:** Formulations used to prepare nanocomposite hydrogels.

Description	Code
Hydrogel 1: Lap 6%	A1
Hydrogel 2: PEGDA 10%, Lap 6%, UV light	A2
Hydrogel 3: PEGDA 10%, Lap 6%, IG 0.05%, UV light	A3
Hydrogel 4: 10% PEGDA, UV light	A4

**Table 2:** Groups used in in vivo assays.

Group	Description
G1	Control group
G2	Lap 6%
G3	PEGDA 10% + Lap 6%
G4	PEGDA 10% + Lap 6% + IG 0.05%
G5	PEGDA 10%

**Table 3:** Effect of different formulations on histopathological changes in bone tissue during bone repair.

Groups	Fibrocartilage	Endochondral Ossification	Bone Spicules	Intramembranous Ossification
G1	+++	++	+	−
G2	++	++	+	++
G3	++	++	+	+
G4	++	+++	++	++
G5	+	++	++	+

The intensity of changes was assessed semi-quantitatively and is denoted as follows: (−) no changes; (+) mild process; (++) moderate process; (+++) intense process.

## Data Availability

The data presented in this study are available in the manuscript itself.
